# A Chromosome-Level Genome Assembly of Mozambique Tilapia (*Oreochromis mossambicus*) Reveals the Structure of Sex Determining Regions

**DOI:** 10.3389/fgene.2021.796211

**Published:** 2021-12-08

**Authors:** Wenjing Tao, Jianmeng Cao, Hesheng Xiao, Xi Zhu, Junjian Dong, Thomas D. Kocher, Maixin Lu, Deshou Wang

**Affiliations:** ^1^ Key Laboratory of Freshwater Fish Reproduction and Development (Ministry of Education), Key Laboratory of Aquatic Science of Chongqing, School of Life Sciences, Southwest University, Chongqing, China; ^2^ Pearl River Fisheries Research Institute, Chinese Academy of Fisheries Science, Key Laboratory of Tropical and Subtropical Fishery Resource Application and Cultivation, Ministry of Agriculture, Guangzhou, China; ^3^ Department of Biology, University of Maryland, College Park, Rockville, MD, United States

**Keywords:** Mozambique tilapia, genome, sex chromosome, sex determining region, transition

## Abstract

The Mozambique tilapia (*Oreochromis mossambicus*) is a fascinating taxon for evolutionary and ecological research. It is an important food fish and one of the most widely distributed tilapias. Because males grow faster than females, genetically male tilapia are preferred in aquaculture. However, studies of sex determination and sex control in *O*. *mossambicus* have been hindered by the limited characterization of the genome. To address this gap, we assembled a high-quality genome of *O*. *mossambicus*, using a combination of high coverage of Illumina and Nanopore reads, coupled with Hi-C and RNA-Seq data. Our genome assembly spans 1,007 Mb with a scaffold N50 of 11.38 Mb. We successfully anchored and oriented 98.6% of the genome on 22 linkage groups (LGs). Based on re-sequencing data for male and female fishes from three families, *O*. *mossambicus* segregates both an XY system on LG14 and a ZW system on LG3. The sex-patterned SNPs shared by two XY families narrowed the sex determining regions to ∼3 Mb on LG14. The shared sex-patterned SNPs included two deleterious missense mutations in *ahnak* and *rhbdd1*, indicating the possible roles of these two genes in sex determination. This annotated chromosome-level genome assembly and identification of sex determining regions represents a valuable resource to help understand the evolution of genetic sex determination in tilapias.

## Introduction

Sex determination, the process by which a sexually reproducing organism initiates differentiation as a male or female, is triggered either by genetic factors on sex chromosomes or environmental factors (Capel, 2017). Cytological analysis can only distinguish highly divergent sex chromosomes with significant differences in morphology (Wright et al., 2016; Palmer et al., 2019). The sex chromosome systems of mammals and birds are highly conserved, with cytologically differentiated sex chromosomes that have lost many ancestral genes and gained a large number of repetitive sequences. Fishes display a diversity of sex determining systems, from environmental to genetic sex determination (Schartl, 2004; Nagahama, 2005). Most fish have homomorphic sex chromosomes which are indistinguishable by shape (Devlin and Nagahama, 2002; Bachtrog et al., 2014). Thus, the identification and characterization of sex chromosomes in fishes has only advanced in recent years, when advances in DNA sequencing and bioinformatic methods made it possible to identify and characterize these less diverged sex chromosomes. Sex chromosomes have now been characterized in a wide variety of fishes based on both chromosome-level genome assemblies generated from long read sequence data, as well as re-sequencing of male and female genomes using short read sequencing technology (Dixon et al., 2019; Pan et al., 2019; Li et al., 2020; Peichel et al., 2020; Wen et al., 2020; Einfeldt et al., 2021; Li et al., 2021; Nakamoto et al., 2021; Tao et al., 2021; Xue et al., 2021).

Cichlids are an excellent model for understanding the evolution of sex determination as they show unusual diversity of sex chromosome systems (Gammerdinger and Kocher, 2018; El Taher et al., 2021). Several species of tilapias are the economically most important species of cichlids, and they are widely grown in tropical and subtropical climates. In aquaculture, all-male tilapias are favored because of the superior growth rate of males compared to females (Mair et al., 1995; Zhong et al., 2019), and the control of unwanted reproduction during the grow-out period. Therefore, the genetic basis of sex determination in tilapia has been studied for over 50 years. Both male heterogametic XX/XY system and female heterogametic ZZ/ZW system have been identified in tilapia (Cnaani et al., 2008). Variation of sex determining systems is apparent among closely related tilapia species, or even in different populations of the same species. For example, in different populations of Nile tilapia (*Oreochromis niloticus*), male sex determining loci have been identified on linkage groups (LG) 1, 20 and 23 (Gammerdinger et al., 2014; Li et al., 2015; Palaiokostas et al., 2015). An XY system on LG1 has been identified in Stirling Nile tilapia stock and blackchin tilapia (*Sarotherodon melanotheron*) (Gammerdinger et al., 2014; Gammerdinger et al., 2016). Both the closely related blue tilapia (*O*. *aureus*) and more diverged spotted tilapia (*Pelmatolapia mariae*) segregates an epistatically dominant ZW locus on LG3 (Conte et al., 2017; Gammerdinger et al., 2019; Tao et al., 2021). Recently, *banf2w* has been found to be concordant with female determination in four tilapias with LG3 ZW/ZZ SD-systems, including *O*. *aureus*, *O*. *tanganicae*, *O*. *hornorum* and *P*. *mariae* (Curzon et al., 2021). The major sex-determination gene on LG23 is a duplication of *amh*, which is known to play an important role in the vertebrate sex-determination network (Li et al., 2015; Pan et al., 2021b). The sex determining systems of other species in this lineage, including the Mozambique tilapia (*O*. *mossambicus*), remain to be elucidated.

The Mozambique tilapia is native to inland and coastal waters in southeastern Africa. It has been introduced to many tropical and subtropical habitats in the world, and plays a significant role in fish production around the world (El-Sayed, 2019) due to its adaptability to brackish water. Microsatellite DNA markers on LG1 and LG3 are found to be associated with sex based on QTL studies from the F_2_ of an interspecific cross between *O*. *aureus* and *O*. *mossambicus* (Cnaani et al., 2003; Cnaani et al., 2004). Recently, comparison of re-sequencing data of *O*. *mossambicus* against the reference genome of *O*. *niloticus* revealed a high density of XY-patterned SNPs uniformly spread across the first 10 Mb of LG14 (Gammerdinger et al., 2019). This large differentiated sex determining region might be explained by either error in the anchoring process or genome differences between *O*. *niloticus* and *O*. *mossambicus*. Thus, a chromosomal level genome assembly of *O*. *mossambicus* would facilitate the identification of sex-determining regions and further characterization of the sex determining genes in this species.

In the present study, we generated a chromosome-level genome assembly of a female *O*. *mossambicus*. We identified sex determining regions on LG14 and LG3 based on the re-sequencing data of different families, and compared the sex determining region on LG14 identified from two families. Our study lays a foundation for understanding the genetic basis of sex determination in Mozambique tilapia.

## Materials and Methods

### Ethics Statement

Animal experiments were conducted in accordance with the regulations of the Guide for Care and Use of Laboratory Animals and were approved by the Committee of Laboratory Animal Experimentation at Southwest University.

### Sample Collection and Sequencing

To generate a chromosome-level assembly of *O*. *mossambicus*, a whole XX female fish was sourced from Pearl River Fishery Research Institute in 2019. Phenotypic sex was determined based on histological observation of gonad tissue as described (Tao et al., 2018). Skeletal muscle was flash frozen in liquid nitrogen and the DNA was extracted using a Blood and Cell Culture DNA Midi Kit (Q13343, Qiagen) to construct Illumina and Nanopore library. To identify a full range of transcripts, gill, brain, heart, kidney, head kidney, muscle and testis were collected and stored in RNAlater.

An improved Hi-C procedure (Rao et al., 2014) was adapted for Hi-C library construction. Briefly, the blood sample was treated with 1% formaldehyde for 10 min at room temperature and the reaction was quenched by adding glycine. Nuclei were digested with 100 units of DpnII, labeled with biotin-14- dCTP (Invitrogen), and ligated using T4 DNA ligase. Subsequently, the ligated DNA was sheared into fragments of 300–600 bp. These Hi-C libraries were sequenced on the Illumina HiSeq X Ten platform.

Seven RNA samples were extracted from gill, brain, heart, kidney, head kidney, muscle and testis using Trizol (Invitrogen) according to the manufacturer’s instructions. The RNA was treated with DNaseI (RNase-free, 5 U μl-1) to remove DNA contamination. Poly-T Oligonucleotides attached to magnetic beads were used to isolate poly(A) mRNA from total RNA according to the Illumina sample preparation guide. Paired-end cDNA libraries of different tissues were constructed using the RNASeq NGS library preparation kit (Gnomegen) and sequenced on the Illumina HiSeq 2500 system with a read length of 150 bp.

### Genome Assembly and Synteny Analyses

To generate a high-quality genome assembly, we generated Nanopore long reads (110.57 Gb), 10x Genomics Illumina reads (30.42 Gb), and Hi-C Illumina read pairs (104.66 Gb). Oxford Nanopore basecalling was performed using Guppy V3.2.2 (Wick et al., 2019). A performance-oriented assembler NextDenovo V2.0-beta.1 (https://github.com/Nextomics/NextDenovo) with default parameters was used for genome assembly, including sequencing error correction, preliminary assembly, and genome polishing (Supplementary Figure S1). The NextCorrect module was used to correct sequencing errors and extract consensus sequence. These qualified long reads were assembled to obtain the preliminary assembly by NextGraph. Genome polishing to fix base errors was performed using NextPolish. Subsequently, the Hi-C data was processed to assemble the chromosomal-level genome. For the Illumina reads of HiC sequencing, fastp (Chen et al., 2018) was used to remove low quality reads, trim adapters and polyG tails. The clean Hi-C reads were aligned to the assembled scaffolds using bowtie2 V2.3.2 (Langmead and Salzberg, 2012) with the end-to-end model (-very- sensitive -L 30). The contact read count among each scaffold was calculated and normalized by standardizing the digestion sites of *DpnII* on the genome sketch. Then LACHESIS (Burton et al., 2013) was used to order and orientate the scaffolds. Finally, Juicebox (Durand et al., 2016) was used to visualize and manually adjust the candidate assembly until the overall Hi-C interaction heatmap conformed to the overall features of chromosome. Synteny analyses among *O*. *mossambicus*, *O*. *niloticus* and *Archocentrus centrarchus* were carried out using MUMmer (Delcher et al., 2003) and MCScanX (Wang et al., 2012).

### Genome Annotation

Tandem repeats were identified using the tandem repeats finder (Benson, 1999) and GMATA (Wang and Wang, 2016) with default parameters. Interspersed repeats (transposable elements, TEs) were identified using a combination of *de novo* and homology-based approaches. MITE-Hunter (-n 20 -P 0.2 -c 3) (Han and Wessler, 2010) was used to identify miniature inverted repeat TEs and other small class II nonautonomous TEs. A *de novo* repeat library was constructed by Repeatmodeler V1.0.4 (Flynn et al., 2020) and classified automatedly by TEclass (Abrusan et al., 2009). Finally, Repeatmasker V4.0.6 (Chen, 2004) was adopted to search the repeat regions against the repeat library.

Protein-coding genes were predicted using *ab initio*, homology and RNA sequence-based methods. The *ab initio* gene prediction and gene structures were generated by Augustus v3.3.1 (Stanke et al., 2006). For homology-based gene prediction, protein sequences of *Oreochromis niloticus* (GCF_001858045.2), *Oryzias latipes* (GCF_002234675.1), *Danio rerio* (GCA_000002035.4), *Xiphophorus maculatus* (GCF_002775205.1), and *Astyanax mexicanus* (GCA_000372685.2) were downloaded from NCBI and GeMoMa V1.6.1 (Keilwagen et al., 2016) was adopted with default parameters. For the *de novo* prediction, RNA-seq reads were *de novo* assembled using StringTie (Pertea et al., 2015). PASA (https://github.com/PASApipeline/PASApipeline) was used to produce a unigene set. Finally, EVM v1.1.1 (Haas et al., 2008) was used to integrate the gene models predicted by these three different methods and produce a consensus gene set. Transposonpsi (http://transposonpsi.sourceforge.net) was used to align the gene set to the transposon database with default parameters. Genes containing homology to transposons were removed from the final gene set. BUSCO V5.2.2 (Simao et al., 2015) was used to perform quantitative assessment of genome assembly and measure annotation completeness by searching the conserved single-copy genes across Actinopterygii against the predicted gene models of the assembled genome. Completeness of the genome assembly was also estimated with CEGMA v2.5 (Parra et al., 2007). The functional annotation of the predicted genes of *O*. *mossambicus* were performed by homology searches against public gene databases, including the NR, SwissProt, KOG, KEGG and GO databases using BLAST v2.2.31 (Altschul et al., 1990). The results from searches against these 5 databases were concatenated as the final annotation result. For the noncoding RNAs annotation, different methods were adopted. The rRNA, snRNA and miRNA sequences were predicted by Infenal 1.1 (Nawrocki and Eddy, 2013) on Rfam (Griffiths-Jones et al., 2003) and miRBase (Kozomara et al., 2019). The rRNA sequences and their subunits were annotated using RNAmmer V1.2 (Lagesen et al., 2007). The tRNA sequences were predicted by tRNAscan-SE v1.3.1 (Lowe and Eddy, 1997) with the default parameters.

### Phylogenomic Analyses and Divergence Time Estimation

To identify gene families in the *O*. *mossambicus* genome, genome sequences and annotation files of *O*. *niloticus* (GCF_001858045.2), *O*. *latipes* (GCF_002234675.1), *D*. *rerio* (GCA_000002035.4), *X*. *maculatus* (GCF_002775205.1), *A*. *mexicanus* (GCA_000372685.2), *Takifugu rubripes* (GCF_901000725.2), *Astatotilapia calliptera* (GCA_900246225.3), *Archocentrus centrarchus* (GCF_007364275.1), *Pundamilia nyererei* (GCA_000239375.1), *Maylandia zebra* (GCF_000238955.4), *Neolamprologus brichardi* (GCF_000239395.1), *Haplochromis burtoni* (GCF_018398535.1) and *O*. *aureus* (GCF_013358895.1) were downloaded from NCBI. OrthoMCL V2.0.9 (Li et al., 2003) was used to identify gene families among the genomes of these species. The protein sequences of the selected species were aligned to identify orthologs, paralogs and single-copy orthologs, respectively.

A phylogenetic tree among *O*. *mossambicus* and the representative species was constructed based on the single-copy orthologs. The protein sequences of these single-copy orthologs were concatenated into a supermatrix, which was aligned using Mafft V7.313 (Katoh et al., 2002). The poorly aligned sequences were then eliminated using Gblocks (Castresana, 2000). Phylogenetic analyses were performed using RAxML (Stamatakis, 2006) with the GTRGAMMA model and 1,000 bootstrap replicates. Based on the phylogenetic tree, MEGA-CC (Mello, 2018) was adopted to compute the mean substitution rates and estimate divergent time. Gene family expansion and contraction (gain and loss) were estimated using CAFÉ V4.2.1 (De Bie et al., 2006) over the constructed phylogenetic tree.

### Analyses of Sex-Linked Regions

Re-sequencing data for two families (Family 1: 8 M, 12 F; Family 2: 15 M, 15 F) of *O*. *mossambicus* were downloaded from NCBI (Gammerdinger et al., 2019) and mapped to the newly assembled *O*. *mossambicus* genome. Sex-linked regions were identified as described (Gammerdinger et al., 2019). Briefly, variants were called using GATK V4.2 software package (McKenna et al., 2010). HaplotypeCaller and GenotypeGVCF implemented in GATK were used to obtain a single output VCF file. The alignment files were also converted into mpileup files using Samtools V1.9 (Li et al., 2009) and subsequently into sync files using Popoolation2 (Kofler et al., 2011). Next, each family was processed with Sex_SNP_finder_GA.pl (https://github.com/Gammerdinger/sex-SNP-finder) to identify XY and ZW sex-patterned SNPs, and to calculate FST values. The density of these sex-patterned SNPs was quantified in non-overlapping 10 kb windows and examined for an elongation of the high SNP density tail in the distributions of both the XY- and ZW-patterned SNPs.

To narrow the sex-determining regions, the sex-linked regions of *O*. *mossambicus* were also identified based on individual re-sequencing data as described (Xue et al., 2021). Briefly, genome re-sequencing data of 5 male and 5 female fish from the Pearl River Fishery Research Institute were collected individually. The phenotypic sex of each fish was identified through gonadal histology and DNA was extracted from muscle tissue. Paired-end libraries were constructed with an insert size of 300–500 base pairs (bp) according to manufacturer’s protocol and subsequently sequenced on an Illumina HiSeq X Ten platform. The raw reads were filtered and trimmed using fastp (Chen et al., 2018) and then mapped against the assembled chromosome-level genome using BWA-MEN (Li, 2013) using the default parameters. After marking and removing duplicates with MarkDuplicate implemented in Picard tools (https://broadinstitute.github.io/picard/), single-nucleotide polymorphisms (SNPs) were called using the joint calling pipeline of GATK V4.1.4.0 (Van der Auwera et al., 2013). The parameters “QD < 2.0 || FS > 60.0 || MQRankSum < -12.5 || RedPosRankSum < -8.0 || SOR >3.0 || MQ < 40.0” (Xue et al., 2021) were used to filter the SNPs. SNPeff 4.3t (Cingolani et al., 2012) was used to identify the SNPs that are homozygous in one sex but heterozygous in the other sex. Patterns of sex-linked SNPs were compared to identify the sex determining systems. The VCF files of different families were compared to extract the shared sex-patterned SNPs using Bedtools V2.29.1 (Quinlan and Hall, 2010).

### Functional Annotation

The functional annotation and potential coding properties of sex-linked SNPs was performed using ANNOVAR (Wang et al., 2010) with the annotated gene models. mRNA models without full length protein coding sequences were excluded from the analysis. Missense mutations located in exonic regions were then analyzed to predict the functional impact of the missense mutation using PROVEAN V1.1.5 (Choi and Chan, 2015). Substitutions with scores lower than the recommended PROVEAN threshold of −2.5 were considered to be deleterious. The 3D structure models for genes with deleterious mutation were developed using Phyre2 (http://www.sbg.bio.ic.ac.uk/phyre2) for predicting the protein structure by homology modeling under “intensive” mode.

## Results

### Chromosome-Level Assembly and Genomic Characteristics of *O*. *mossambicus*

**FIGURE 1:**
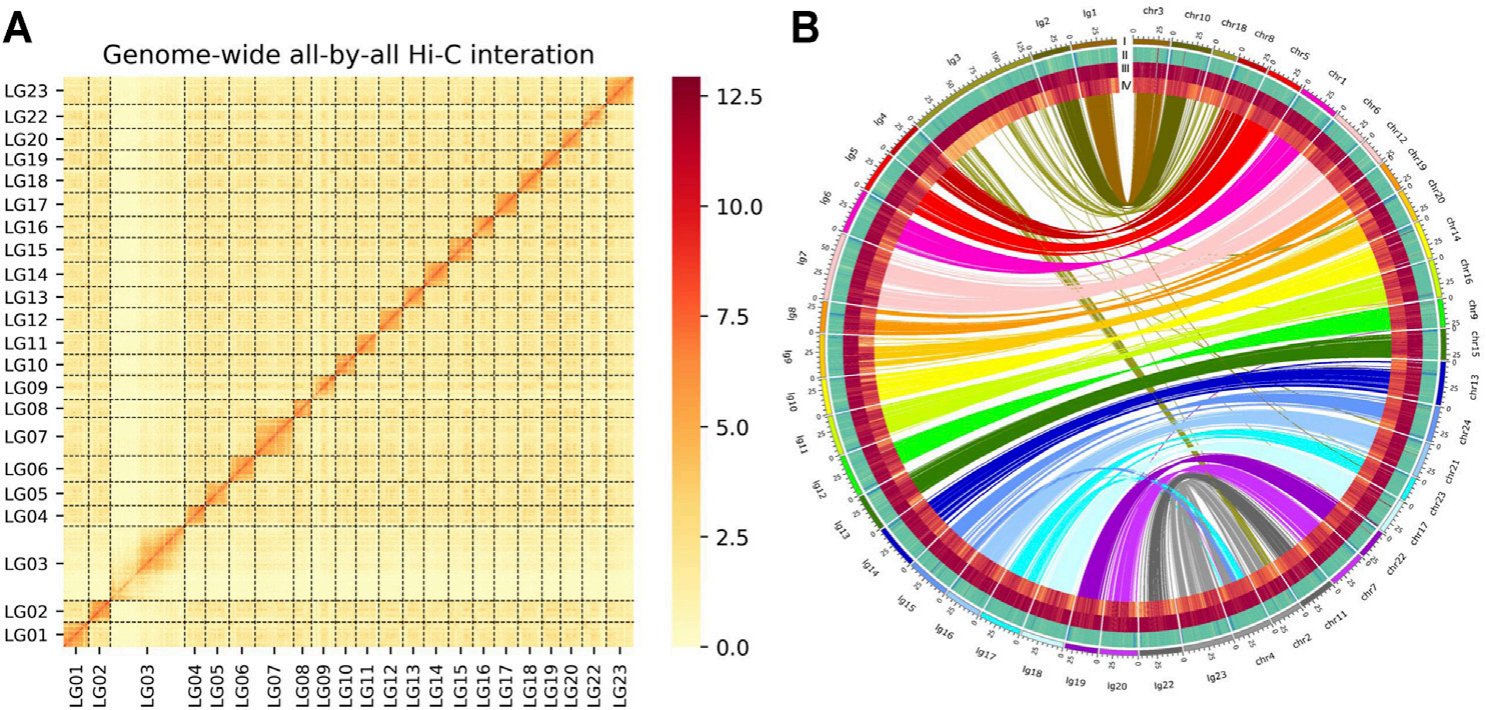
**(A)** Genome-wide chromosomal contact matrix of *O*. *mossambicus* based on chromatin interaction data generated by Hi-C. The low to high interaction frequency distribution of Hi-C links among chromosomes is shown from light yellow to dark red in the heatmap. **(B)** Comparison of the genome of *O. mossambicus* and *A. centrarchus*. A Circos atlas presents details of the pseudo-chromosome information from outside to inside: (I) the length of each chromosome, (II) GC content of 100-Kb genomic intervals, (III) density of gene distribution in each 100-Kb genomic interval, (IV) density of repeats in each 100-Kb genomic interval. A synteny comparison between *O*. *mossambicus* and *A*. *centrarchus* genomes (Chr represents *A*. *centrarchus* and lg represents *O*. *mossambicus*) revealed high accuracy of our assembled *O*. *mossambicus* genome.

We used a combination of Nanopore long reads, Illumina short reads, and Hi-C technologies (Supplementary Figures S2–S3 and Supplementary Table S1), to generate a chromosome-level genome assembly for *O*. *mossambicus*. A total of 110.57 Gb Nanopore long reads were used to assemble a preliminary genome sequence, which was subsequently polished. Additionally, a total of 714 million raw Illumina reads with total length of about 104.66 Gb generated from the Hi-C library were applied to identify contacts among the Nanopore contigs, of which 357 million pairs of clean reads covered 99.93% of the assembled genome. The final assembly had 22 chromosomes with a total length of 1,007 Mb (Table 1), and 98.62% of contigs were anchored to the chromosomes. The heatmap of chromosome contact demonstrates that the genome assembly was complete and robust (Figure 1A). The genome size of *O*. *mossambicus* is comparable to that of its mostly related species, including *O*. *niloticus* and *O*. *aureus*. The contig N50 and scaffold N50 of the *O*. *mossambicus* assembly were 2.78 and 11.38 Mb (Table 1, Supplementary Table S2 and Supplementary Figure S4), respectively. The largest chromosome was LG3 with 130.76 Mb in length containing 46 scaffolds, and the smallest was LG19 with 32.83 Mb containing only 2 scaffolds. *A. centrarchus* is the most closely related species with a high-quality chromosome-scale assembly and a typical teleost karyotype of 24 chromosome pairs. To evaluate the quality of the assembly and facilitate the identification of chromosome fusion, we performed synteny analyses among *O*. *mossambicus*, *O*. *niloticus* and *A*. *centrarchus*. There were 39,689 synteny blocks (>2 kb) between the assembled genomes of *O*. *mossambicus* and *A*. *centrarchus*. Almost all chromosomes showed the 1:1 synteny relationship, with the exception of LG3 in the fish that aligned to Chr2 and Chr18 of *A*. *centrarchus* (Figure 1B). Genome synteny analysis also revealed good collinear relationship of the genome between *O*. *mossambicus* and *O*. *niloticus* (Supplementary Figure S5). The genome assembly of *O*. *mossambicus* had 97.71% complete BUSCO genes and 239 of 248 (96.38%) of conserved core eucaryotic genes in CEGMA v2.5 database (Supplementary Table S3). These results indicated that the genome assembly was of highly completeness. Moreover, nearly a half of the assembled sequences on LG3 showed no detectable synteny with any other chromosomes, supporting the hypothesis of B chromosome fusion origin, instead of autosome fusion origin, of this giant chromosome (Conte et al., 2021).

**TABLE 1:** Summary of representative cichlid genomes at chromosome level.

	*O*. *mossambicus*	*O*. *niloticus*_Eg	*O*. *niloticus*_Jp	*O*. *aureus*	*M*. *zebra*	*A*.*centrachus*	*A*. *citrinellus*	*A*. *calliptera*
Sequencing platform	Nanopore, Hi-C	PacBio	Nanopore, Hi-C	Nanopore, Hi-C	PacBio	PacBio, BioNano, Hi-C	PacBio, BioNano, Hi-C	PacBio, BioNano, Hi-C
Chromosome number	22	22	22	22	22	24	22	22
Assembly size (Mb)	1,007	1,001	1,005	1,006	957.49	961.23	903.07	880.44
GC%	42.28	40.72	41.23	40.65	41.15	39.79	40.59	41.08
Identified genes	28,902	29,537	25,264	25,467	25,898	29,275	-	26,070
Contig	2.78	2.93	2.65	4.40	1.41	2.15	3.84	4.44
N50 (Mb)								
Scaffold	42.17	38.84	40.35	40.72	32.66	35.59	37.15	38,67
N50(Mb)								
References	This study	GCA_001858045.3	GCA_013350305.1	GCA_013358895.1	GCA_000238955.5	GCA_007364235.1	GCA_013435755.1	GCA_900246225.3

Note: A. centrachus indicates Archocentrus centrarchus (flier cichlid), A. citrinellus indicates Amphilophus citrinellus (Midas cichlid), A. calliptera indicates Astatotilapia calliptera (eastern happy), M. zebra indicates Maylandia zebra.

The results of Repbase database and *de novo* prediction showed that repeated sequences accounted for 42.28% of the *O*. *mossambicus* genome, which is similar to *O*. *niloticus* (40.4%), *O*. *aureus* (39.4%) and *O*. *latipes* (42.83%), but lower than *D*. *rerio* (63.12%). Transposable elements accounted for the major parts of repeated sequences in the *O*. *mossambicus* genome. DNA transposons (21.13%) were the most common, followed by LINEs (long interspersed repeated segments, 10.13%) and LTR (long terminal repeats, 6.56%) (Supplementary Table S4). The most abundant tandem repeats were centromeric repeats which were identified on all LGs except LG22 (Supplementary Table S5), including the known tilapia centromeric repeat SATA (Franck et al., 1992). In total, 28,902 protein-coding genes were predicted by *ab initio*, homologous prediction and RNA-seq prediction in the *O*. *mossambicus* genome, with an average gene length of 16,519 bp. The average coding sequence length and intron length were 1,574 bp and 1,733 bp, respectively, with average exon number of 9.62 per gene. The functions of the protein-coding genes were annotated using the NR, TrEMBL, KOG, KEGG, and GO databases. A total of 4,524 tRNAs, 556 rRNAs and 1,170 miRNAs were predicted by Infernal and Rfam databases.

### Phylogenomic Analyses

**FIGURE 2:**
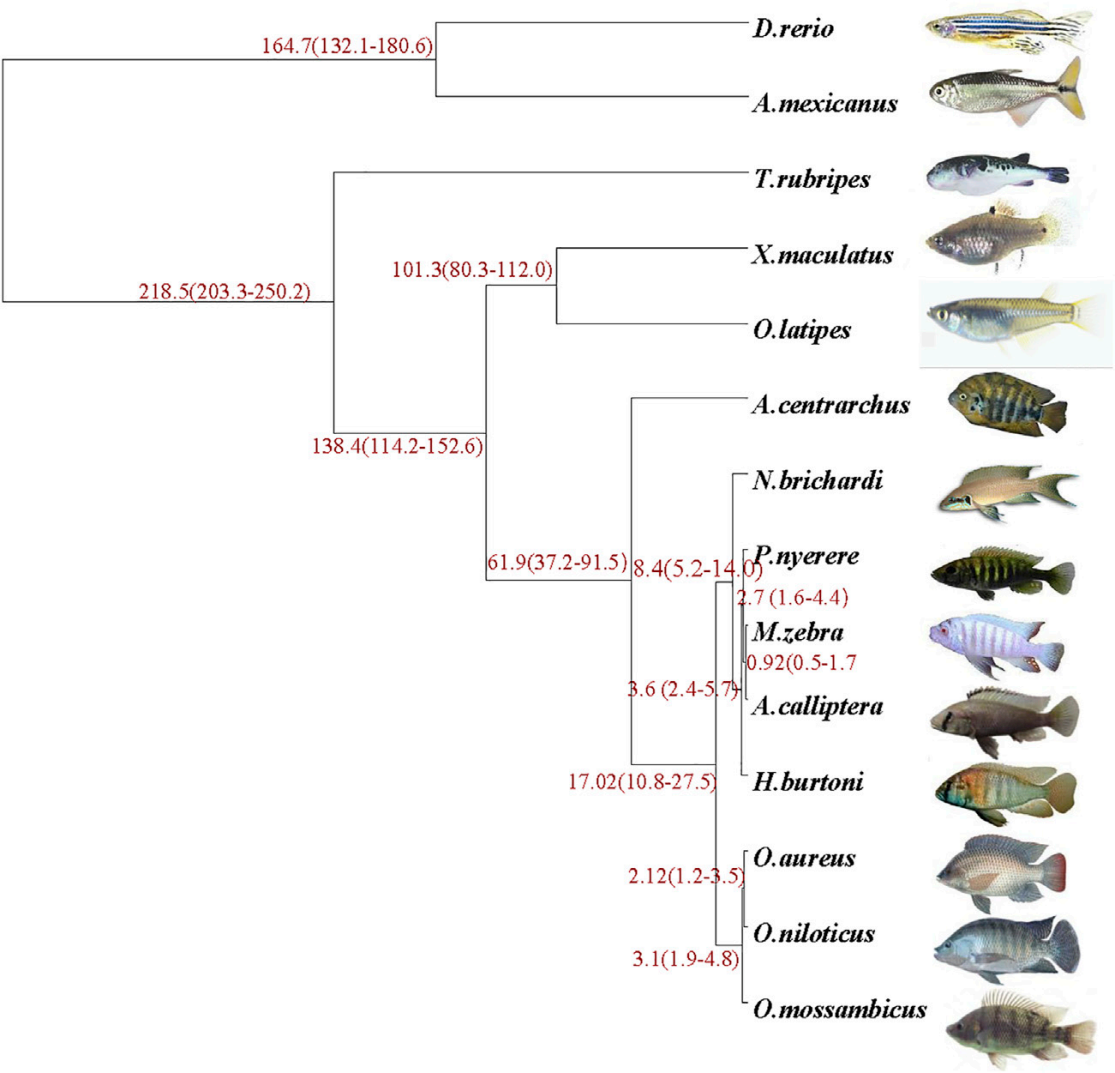
Phylogenetic analysis of *O*. *mossambicus* and other representative teleost fish species. The species divergence time is shown at the branches of the phylogenetic tree, and the confidence intervals are given in parentheses.

Genomes of the *O*. *mossambicus* and other 13 representative fishes (*D*. *rerio*, *A*. *mexicanus*, *T*. *rubripes*, *X*. *maculatus*, *O*. *latipes*, *A*. *calliptera*, *A*. *centrarchus*, *P*. *nyererei*, *M*. *zebra*, *N*. *brichardi*, *H*. *burtoni*, *O*. *aureus* and *O*. *niloticus*) were analyzed to establish the phylogenetic relationship. It appeared that *O*. *mossambicus* was most closely related to the branch consisting of *O*. *aureus* and *O*. *niloticus* (Figure 2). Consistent with previous studies based on phylogenomic analyses (Salzburger, 2018; Ronco et al., 2021), our results showed that cichlids were characterized by exceptionally fast diversification rates. All the nodes displayed 100 confidence intervals, indicating that this tree is robust. The overall topology is consistent with the previously comprehensive phylogenetic work on cichlids and ray-finned fishes (Hughes et al., 2018; Matschiner et al., 2020).

### Identification of Sex Determining Regions

**FIGURE 3:**
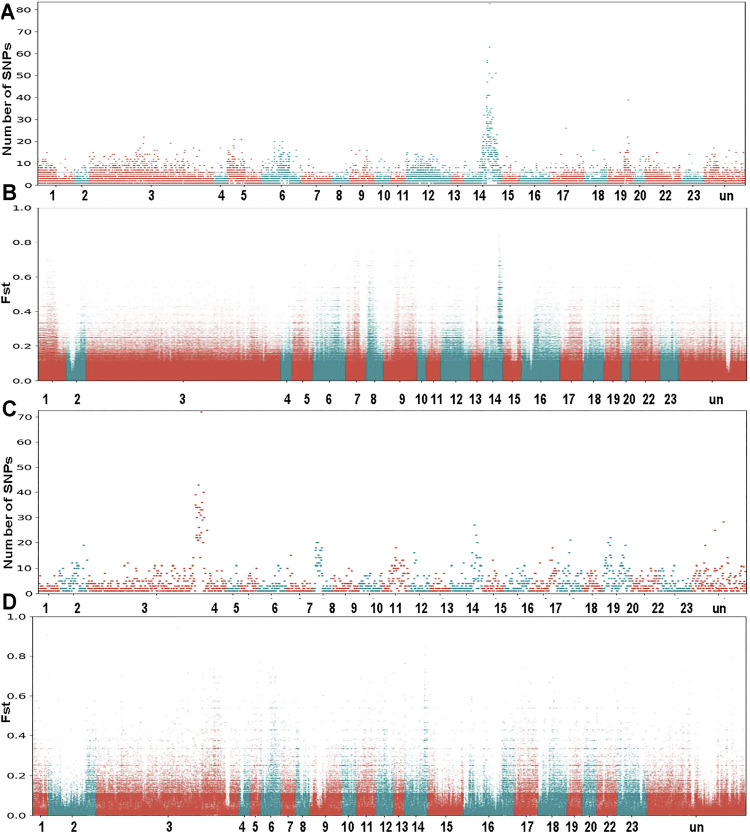
Whole genome survey of sex chromosomes in *O. mossambicus*. **(A)** Sex-patterned variants of Family 1, intermediate frequency SNPs in males that are fixed or nearly fixed in females. **(B)** FST comparison of female pool versus male pool from Family 1. **(C)** Sex-patterned variants of Family 2. **(D)** FST comparison of female pool versus male pool from Family 2. The genome re-sequencing data are retrieved from NCBI SRA database (Gammerdinger et al., 2019).

The *O*. *mossambicus* genome assembly was used to identify sex-determining regions of this species. Based on the whole genome re-sequencing data of pooled DNA from males and females of two families, a novel XY sex determining system on LG14 was characterized in *O*. *mossambicus* (Gammerdinger et al., 2019). We realigned these re-sequencing data to the *O*. *mossambicus* genome assembly and identified the variants that were fixed in either the female pool or the male pool. For Family 1, examination of the whole genome Fst plot comparing males and females revealed a strong signal on LG14 (Figure 3A). This region overlapped with the previously identified XY sex-determining regions in *O*. *mossambicus* (Gammerdinger et al., 2019) which spanned approximately 10 Mb. A total of 12,397 SNPs in Family 1 of *O*. *mossambicus* fitted the sex-patterned criteria. In Family 1, there were 451 non-overlapping 10 kb windows with at least 10 XY-patterned SNPs (Supplementary Table S6). The highest densities of sex-patterned SNPs (with more than 20 sex-linked SNPs per 10 Kb) occurred between 33.3 and 41.8 Mb on LG14 (Figure 3B). LG14 showed comparable repeat content of autosomes based on the Repeat element annotation (Supplementary Table S5). For Family 2, examination of the Fst plot identified a ZW sex determining pattern with strong signals on the giant chromosome LG3 (Figure 3C). A total of 2,290 SNPs in Family 2 fit the ZW-patterned criteria. There were 136 non-overlapping 10 kb windows with at least 10 sex-patterned SNPs in Family 2 (Supplementary Table S7). The highest densities of sex-patterned SNPs (with more than 20 sex-linked SNPs per 10 Kb) occurred between 97.1 and 110.7 Mb (Figure 3D). Our results indicated that multiple sex determination systems have been observed in *O*. *mossambicus*.

**FIGURE 4:**
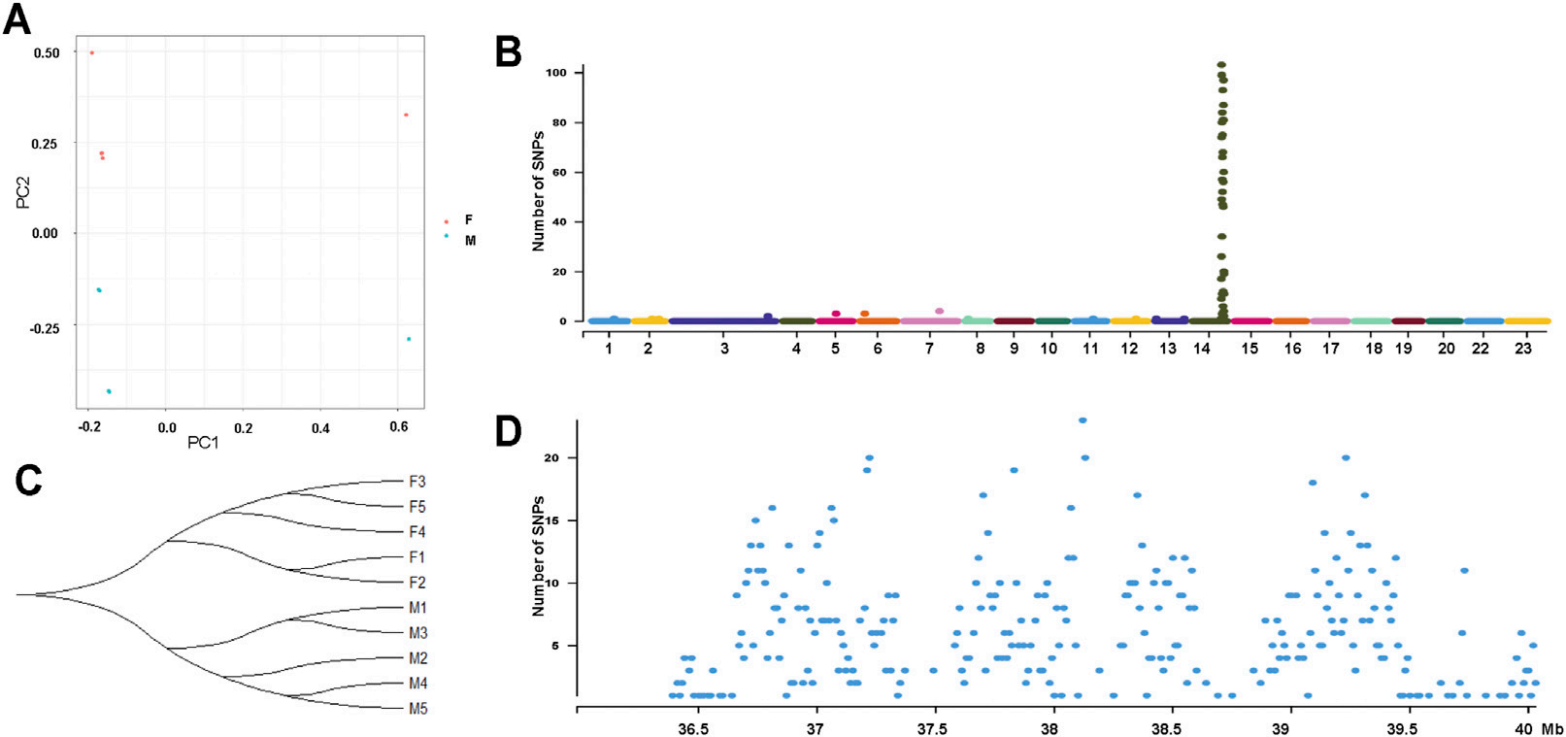
Genome-wide identification of SNPs from 5 male and 5 female individuals from Zhujiang population of *O*. *mossambicus*. **(A)** Principal component analysis of 10 individuals using SNPs. **(B)** Phylogenetic tree showing relationships of male (M1-M5) and female (F1-F5) SNPs. **(C)** Sex-linked SNPs of 10 individuals indicate LG14 is the sex chromosome of the Zhujiang population. **(D)** Density of sex-linked in each 10-Kb genomic interval on LG14.

**FIGURE 5:**
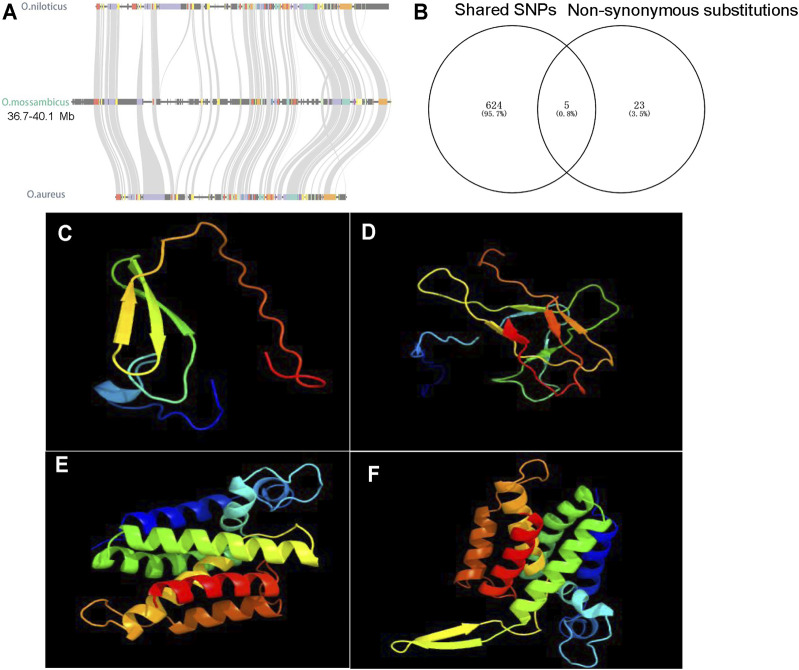
Characterization of sex-determining regions. **(A)** Syntenic relationships of sex-determining regions on LG14 among *O*. *niloticus*, *O*. *aureus* and *O*. *mossambicus*. **(B)** 5 shared non-synonymous substitutions, and the other 23 non-synonymous substitutions are only found in the Zhujiang population. **(C)** The predicted protein structures of Y-linked *ahnak*. **(D)** The predicted protein structures of X-linked *ahnak*. **(E)** The predicted protein structures of Y-linked *rhbdd1*. **(F)** The predicted protein structures of X-linked *rhbdd1*.

To further characterize the degree of sequence differentiation between sexes and identify sex-determining regions, we also sequenced 5 male and 5 female fish individually to an average depth of 20-fold. Using the assembled *O*. *mossambicus* genome as a reference, we detected a total of 4,231,342 SNPs and 1,680 XY-patterned SNPs (Supplementary Table S8). Principal component analysis (Figure 4A) and an SNP tree (Figure 4B) showed that male and female genomes clustered into two distinct groups, indicating sex-specifically differentiated genomic regions. Higher SNP density and substantially higher heterozygosity were presented in male individuals. Moreover, SNP density mapping showed that only 1.23% of the putatively sex-linked SNPs were located on autosomes. The highest SNP density was on the sex chromosome LG14 (Figure 4C). The region from 36.3 to 40 Mb on LG14 displayed high density of sex-patterned SNPs (Figure 4D). No differences in sequencing depth ratio were observed between females and males in this region. Synteny relationships in this region were shared among *O*. *mossambicus*, *O*. *aureus* and *O*. *niloticus* (Figure 5A). These results suggested that LG14 was possibly a young sex chromosome without accumulation of a large number of structural variations (Gammerdinger et al., 2019). To narrow the sex determining region of *O*. *mossambicus*, we compared the sex-linked SNPs identified in the individual re-sequencing with the pooled re-sequencing data from Family 1 (Figure 5B). We found that 629 out of 1,680 sex-patterned SNPs spanning 36.3–40 Mb on LG14 were shared by these two families (Supplementary Table S9). The density of sex-patterned SNPs was highest between 37–38 Mb on LG14 (Supplementary Table S10). We evaluated the location of these sex patterned SNPs and found that only 14 (2.05%) SNPs were located on the exonic regions, while 282 (44.8%) and 305 (48.5%) of the SNPs were located in the intronic and intergenic regions, respectively. Among the 14 SNPs in the exonic regions, 6 were located between 37–38 Mb, in which 3 were harbored on the *ahnak* gene. The functional annotation of the 14 SNPs in the exonic regions showed that 5 missense mutations were located in the *ahnak*, *sae1*, *znf235*, *rhbdd1* and an uncharacterized gene 98.31% identical to LOC106098495. The mutations in *ahnak* and *rhbdd1* were predicted to be deleterious. Protein modelling of *ahnak* and *rhbdd1* using Phyre2 showed that the mutation on the Y allele changed the predicted protein structures of both genes (Figures 5C–F).

## Discussion

Like other tilapias, male Mozambique tilapia grow faster and are more uniform in size than females. For this reason, production of monosex, genetically male populations are desirable for aquaculture. In order to characterize the sex determining system in this species, we developed a highly accurate and contiguous genome assembly. This genome sequence will contribute to the development of technologies to produce all male populations of *O*. *mossambicus* for commercial aquaculture, and may also help us to better understand the unusual diversity of sex chromosomes in tilapias.

### Genome Assembly

A variety of metrics confirm the high quality of our genome assembly for *O*. *mossambicus*. First, we compared the overall size of our assembly to the assembled genome size of closely related species of *Oreochromis*. The genome assembled from 44x PacBio reads from a female *O*. *niloticus* Egyptian strain was 1,001 Mb (Conte et al., 2017). Recent genome assemblies from a female *O*. *niloticus* Japanese strain and male *O*. *aureus* Wuxi strain generated from 100X Nanopore reads by us were 1,005 Mb and 1,006 Mb, respectively (Tao et al., 2021). In the present study, the assembled genome of a female *O*. *mossambicus* is 1,007 Mb, which matches the assembled genome size of sister species in *Oreochromis* genus, and within the range of assembled genome sizes of other cichlids (880–1,007 Mb). Second, standard measures of assembly contiguity, such as contig N50 and scaffold N50, are comparable to those of other cichlids with high quality genome assemblies (Conte et al., 2017; Conte et al., 2019; Tao et al., 2021). Third, measures of annotation completeness based on the CEGMA (Parra et al., 2007) and BUSCO (Simao et al., 2015) gene lists suggest our assembly includes a high proportion of the expected gene content. Fourth, the repeat-rich regions, such as centromeres, were identified on each chromosome except LG22 in the assembled genome. Finally, the assembled giant chromosome (LG3) of *O*. *mossambicus* showed syntenic blocks with Chr2 and Chr18 of *A*. *centrarchus* and the rest of LG3 showed undetectable synteny with other chromosomes of *A*. *centrarchus*, which supports the previous hypothesis of ancient chromosome fusion events of LG3 in the tribe Oreochromini based on the cytogenetic analysis and comparative genomic study (Chew et al., 2002; Conte et al., 2021). Thus, we have assembled a high-quality, chromosome-level genome of *O*. *mossambicus*, which lays the foundation for subsequent identification of sex chromosomes and sex determining regions.

### Sex Determination Regions

Research on sex determination in tilapia has important economic implications because of the different growth rates between sexes, and therefore research on the molecular basis of sex determination in tilapia has been conducted for decades. *Oreochromis niloticus* has one of the best-documented sex determination systems, with major sex determining regions characterized on LG1, LG20 and LG23 (Lee et al., 2003; Li et al., 2015; Palaiokostas et al., 2015; Taslima et al., 2021). Recently, sex determining systems have been characterized in several other tilapias, including *S*. *melanotheron*, *O*. *aureus*, *O*. *mossambicus*, *C*. *zillii* and *P*. *mariae*, by Illumina re-sequencing (Gammerdinger et al., 2016; Conte et al., 2017; Gammerdinger et al., 2019). However, the sequence divergence between these species and the *O*. *niloticus* reference genome used for mapping the reads possibly introduces problems in the analysis. Thus, in the present study, whole genome re-sequencing data of male and female fish from two families of *O*. *mossambicus* (Gammerdinger et al., 2019) has been re-analyzed using our new genome assembly. A high density of sex-linked SNPs is identified on LG14 and LG3 in Family 1 and 2, respectively. Previously, only LG14 was identified as the sex chromosome (Gammerdinger et al., 2019). This discrepancy can be attributed to the different analysis strategies adopted. We analyzed the re-sequencing data of Family 1 and Family 2 independently, while the previous study combined the data of the two families into one dataset. The strong signal on LG14 might override the relatively weak signal on LG3 in the previous study (Gammerdinger et al., 2019). Similar to *O*. *niloticus*, multiple sex determination systems may exist within and among different families of *O*. *mossambicus*. Recently, a duplicate of *banf2* (W-copy) on LG3 has been found to be concordant with female phenotypic sex in *O*. *aureus*, *O*. *tanganicae*, *O*. *hornorum* and *P*. *mariae* with ZW sex determining system (Curzon et al., 2021). However, the W-specific *banf* was not identified in the female fish of Family2 with ZW sex determining system based on the re-sequencing data, suggesting the existence of a different female sex determiner in *O*. *mossambicus*. These results are in sharp contrast to the sex determining systems shared among several closely related species in other fish lineages, for example, the male-specific *amhy* in 11 Esociformes species (Pan et al., 2021a) and *amhr2y* in two seadragons (Qu et al., 2021). A rapid turnover of sex chromosomes and sex-determining genes may have contributed to the adaptative radiation in tilapia and other cichlids (Ser et al., 2010; Ronco et al., 2021).

Although pool-seq data of high genome coverage is a practical method for identifying genes of major effect (Micheletti and Narum, 2018), individual sequencing has been proved to be more efficient in most cases (Cutler and Jensen, 2010). In the previous study, using the genome of *O*. *niloticus* as the reference, a recent genome scan from two families of *O*. *mossambicus* revealed a high density of XY-patterned SNPs uniformly spread across 10 Mb of LG14 (Gammerdinger et al., 2019). In the present study, re-analysis of the same re-sequencing data of Family 1 on the new genome assembly confirmed the same sex-linked region on LG14. However, based on the individual sequencing data of 5 females and 5 males, we successfully narrowed the sex determination region on LG14 to 3.7 Mb (36.3–40.0 Mb). Therefore, comparing SNP density between multiple males and females individually is a powerful approach to identify sex determining regions.

The conserved synteny of LG14 among different tilapias, together with the small sex determining region, suggests that recombination suppression has not spread very far from the sex-determining locus, and structural variants have not yet accumulated. These results suggest that LG14 is an evolutionarily young sex chromosome, consistent with the conclusion based on the lack of a bimodal distribution in the distribution of non-overlapping sex-patterned windows (Gammerdinger et al., 2019). LG14 has not been frequently identified as the sex chromosome of the cichlid fishes from African Lake Tanganyika (El Taher et al., 2021). To our knowledge, LG14 has only been identified as sex chromosomes in lab strains and one natural population of *Astatotilapia burtoni* with polygenic sex determination system (Bohne et al., 2016; Roberts et al., 2016). The broad region of differentiation in *A*. *burtoni* makes it difficult to know if the same genes might be involved in sex determination.

SNPs leading to deleterious substitutions were found in *ahnak* and *rhbdd1*. It is well documented that germ cell number is an important factor in fish sex determination (Uchida et al., 2004; Matsuda, 2005; Fujimoto et al., 2010; Dai et al., 2021). In tilapia, germ cells of female fish continued to proliferate, whereas the germ cell numbers did not change in males at the key stage of sex determination, indicating that germ cell proliferation could also be a critical threshold of sex determination (Kobayashi et al., 2008; Baroiller et al., 2009). It is well documented that TGF-β signaling pathway, the most frequently used pathway in fish sex determination, plays important roles in modulating the germ cell proliferation in the bipotential gonad (Kikuchi and Hamaguchi, 2013; Pan et al., 2021b). *ahnak* was a well-established inhibitor of TGF-β signaling, downregulating cyclin D1/D2 and inhibiting cell growth (Lee et al., 2014). It was considered as one of the candidate genes for sex determination in *O*. *mossambicus* (Gammerdinger et al., 2019). *rhbdd1* is a proapoptotic member of the Bcl-2 family, and its knock-down results in enhanced apoptosis in HEK 293T cells (Wang et al., 2008). Consistently, *rhbdd1* was proved to regulate spermatogonia apoptosis in mouse (Wang et al., 2009). Thus, the deleterious mutations and changed protein structures predicted by PROVEAN and Phyre2 in either *ahnak* or *rhbdd1* could possibly affect the germ cell development and sex determination in *O*. *mossambicus*. The expression data in gonads at the key stage of sex determination and differentiation is important for the identification of sex determiner. Thus, the sequence of a LG14 Y and expression data at the early stages are required to better understand the structure of this sex chromosome and identify sex determining gene in *O*. *mossambicus*.

In conclusion, we generated a chromosome level genome assembly of *O*. *mossambicus*. Two sex determining regions harboring a high density of sex-linked SNPs on LG14 (XY) and LG3 (ZW) were identified in *O*. *mossambicus* based on whole genome re-sequencing data. Our results provide additional support to the idea that sex chromosome transitions occur at a rapid rate in *Oreochromis* fishes.

## Data Availability

The datasets presented in this study can be found in online repositories. The names of the repository/repositories and accession number(s) can be found in the article/Supplementary Material.
