# Supplementary material for: A Chromosome-Level Genome Assembly of Mozambique Tilapia (Oreochromis mossambicus) Reveals the Structure of Sex Determining Regions
Source: Front Genet. 2021 Dec 8;12:796211. doi: 10.3389/fgene.2021.796211 (PMC8692795; doi:10.3389/fgene.2021.796211)
Supplement: Supplementary file 1 [file DataSheet1.docx]

# **Supplementary Figures**


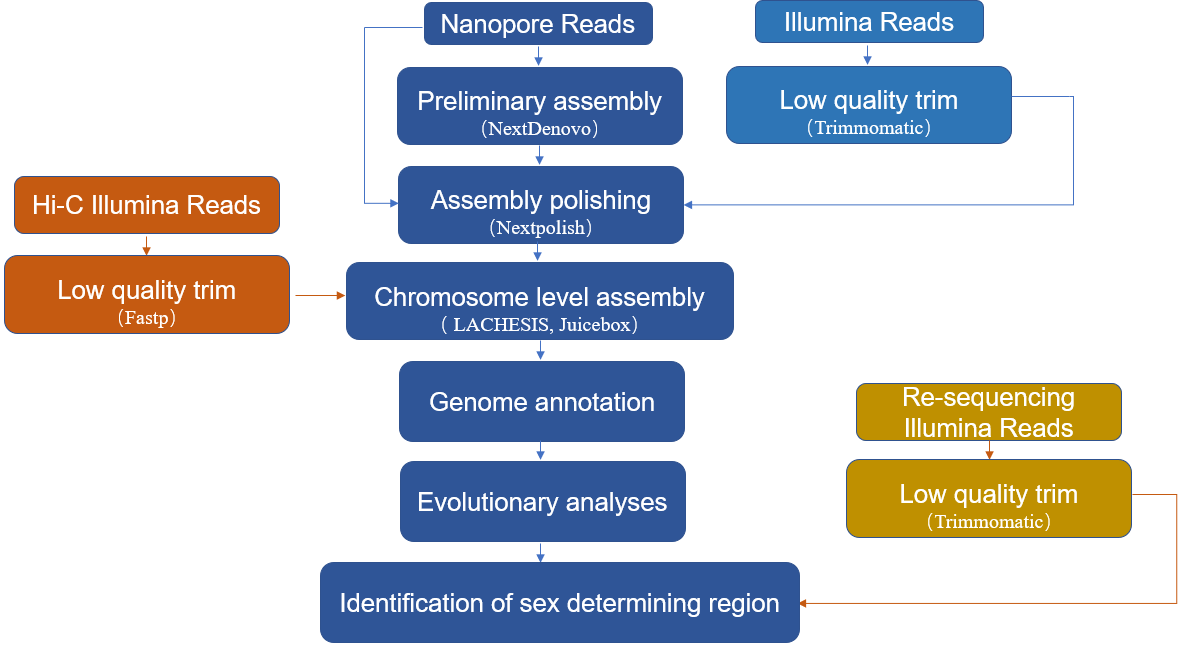


## **Figure S1.** Pipeline for genome assembly and analyses.


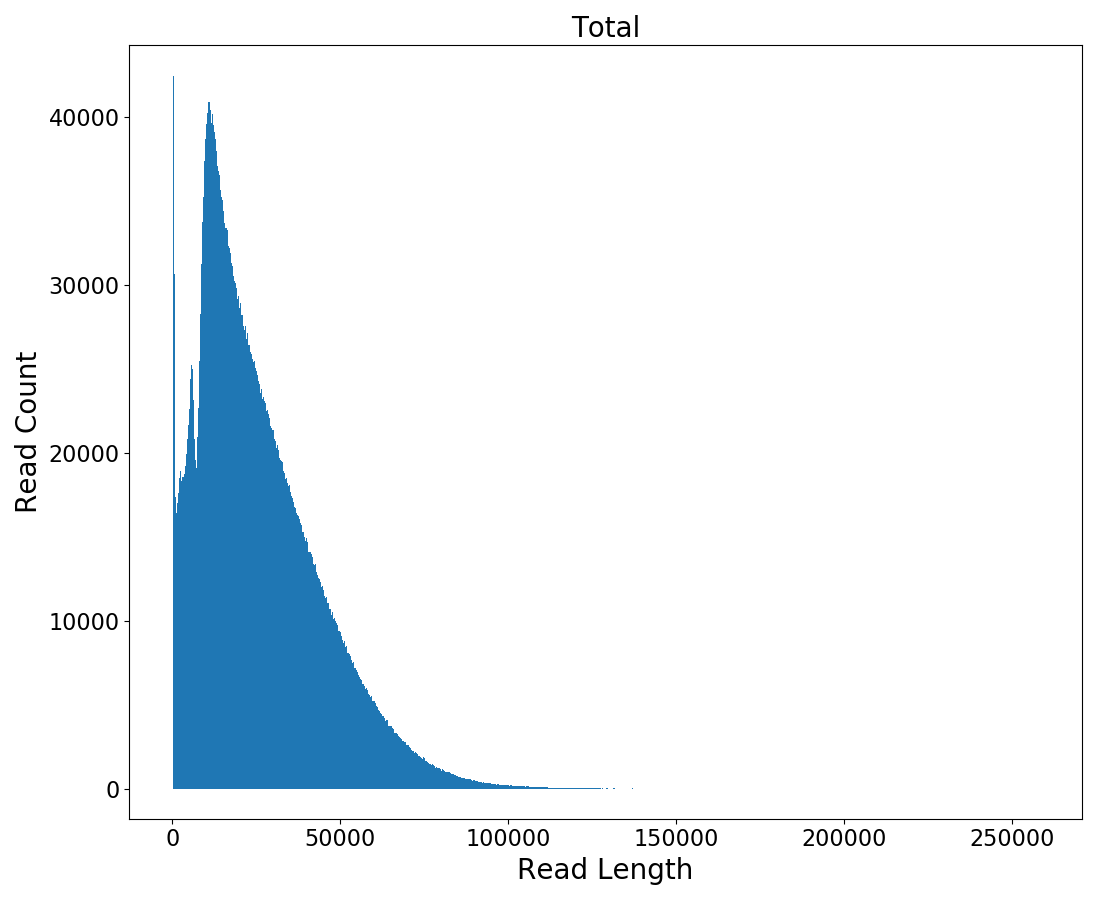


## **Figure S2.** Cumulative length distribution of the Nanopore reads in all runs. The N50 of reads is 36,270 bp.


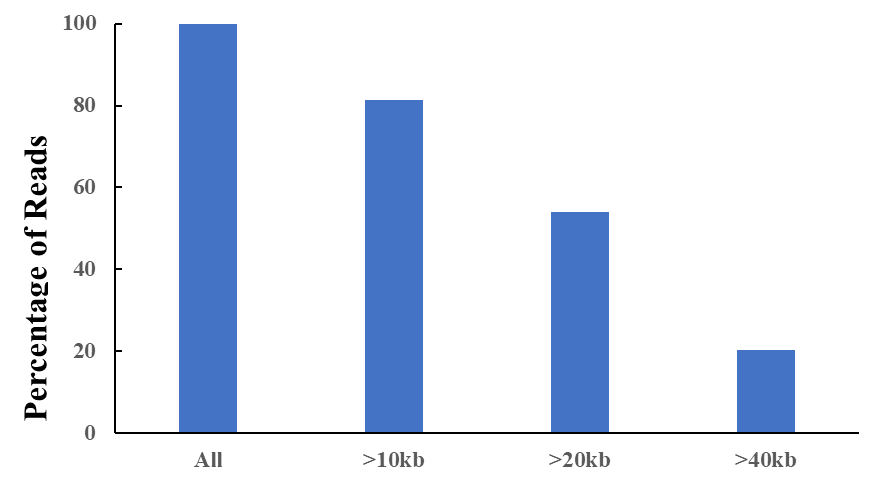


## **Figure S3.** Length distribution of Nanopore reads showing percentages of reads with 10, 20, 30 and 40 kb. 81.39% of all produced reads >=10 kb for *O. mossambicus*.

## **
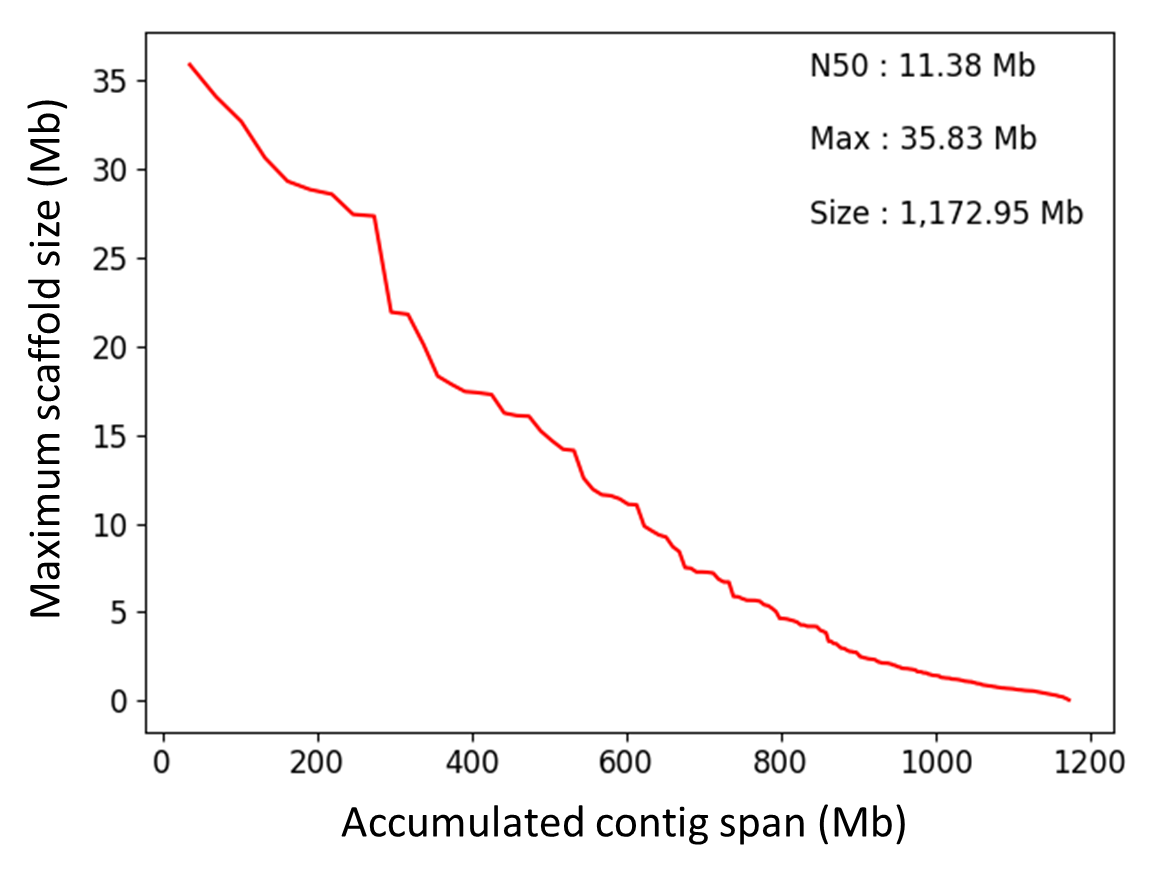
**

## Figure S4. Scaffold length distribution of *O.mossambicus* genome.

##
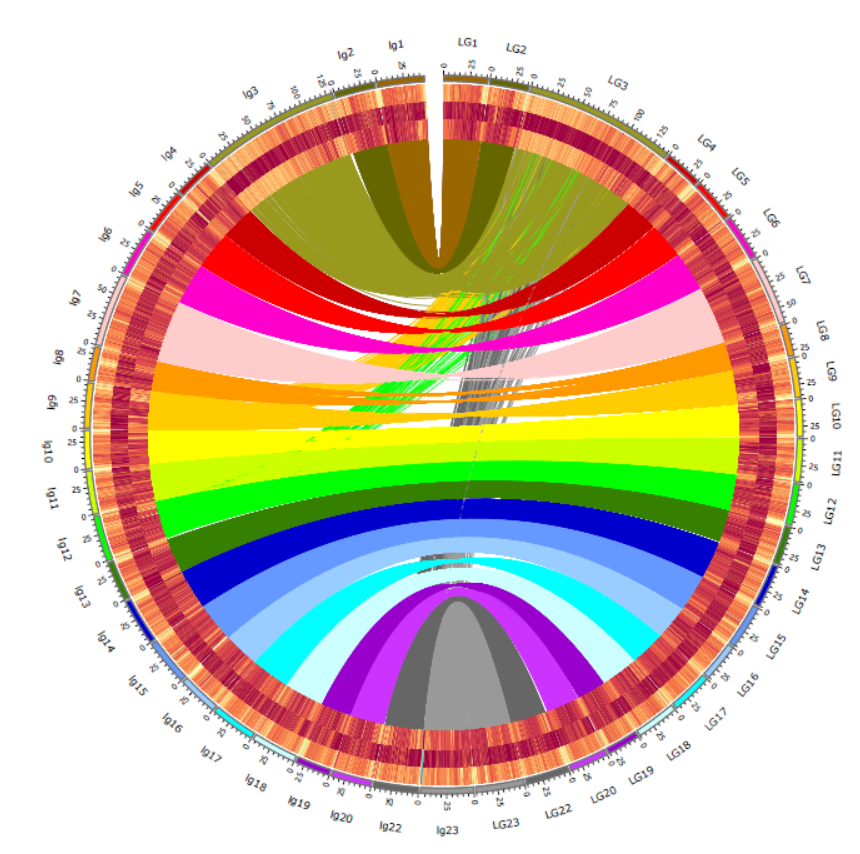


## Figure S5. A synteny comparison between *O. mossambicus* and *O. niloticus* genomes (LG represents *O. niloticus* and lg represents *O. mossambicus*) revealed high accuracy of our assembled *O. mossambicus* genome.
